# Hypercalcaemia in gastrointestinal stromal tumour and sarcoidosis: a case report

**DOI:** 10.1186/s12882-024-03655-2

**Published:** 2024-07-19

**Authors:** Babitha Selvananthan, Eddy Fischer, Raymond Lin

**Affiliations:** 1 Department of Renal Medicine, Nepean Hospital, Sydney, NSW Australia; 2 Sydney Medical School, University of Sydney, Sydney, NSW Australia

**Keywords:** Hypercalcaemia, Sarcoidosis, Gastrointestinal stromal tumour, Acute kidney injury, Case report

## Abstract

**Background:**

Hypercalcaemia is a common manifestation of sarcoidosis but is sparingly described in gastrointestinal stromal tumours (GISTs). We describe a case of acute kidney injury and hypercalcemia resulting from simultaneous diagnosis of GIST and sarcoidosis, the presentation of which has not yet been reported.

**Case Presentation:**

A 61-year-old male presented with acute kidney injury and hypercalcemia, with elevated 1,25-dihydroxyvitamin D levels. Investigations demonstrated a large gastric antral mass which was resected and proven to be GIST. Histopathology of incidentally found liver nodules revealed non-necrotising epithelioid granulomas consistent with concomitant sarcoidosis. The hypercalcemia was successfully treated with bisphosphonate therapy, resection of the GIST and a four month course of corticosteroids, which was truncated due to a mycobacterial infection.

**Conclusions:**

Our case report is the first to describe hypercalcemia due to GIST and biopsy-proven sarcoidosis, thereby raising the possibility of a common pathophysiological pathway relating the two entities. We review the literature describing the mechanisms of hypercalcaemia in GIST and the association between GIST and sarcoidosis.

**Supplementary Information:**

The online version contains supplementary material available at 10.1186/s12882-024-03655-2.

## Background

GISTs are the most common subtype of sarcoma affecting the gastrointestinal tract. Presentation with hypercalcaemia, however, is rare. We report a presentation of GIST with hypercalcaemia and concomitant clinical and histological findings of sarcoidosis, which has not been previously reported. Our case report contributes to the growing number of case reports describing GIST presenting with hypercalcemia and acknowledges a possible relationship between sarcoidosis and GIST.

## Case presentation

A 61-year-old male with Stage IIIA chronic kidney disease (baseline creatinine 130µmol/L) secondary to hypertensive nephrosclerosis, presented with acute kidney injury and hypercalcemia (creatinine 286µmol/L, corrected calcium 3.52mmol/L). He reported polydipsia and polyuria without any constitutional symptoms. He had no early satiety, anorexia or vomiting. His background included hypothyroidism and gastro-oesophageal reflux disease. He was a non-smoker and his regular medications were thyroxine, esomeprazole and metoprolol. Clinical examination revealed splenomegaly without lymphadenopathy, hepatomegaly, synovitis or uveitis.

Investigations demonstrated an appropriately suppressed parathyroid hormone (PTH) level of 1.2pmol/L (1.6-7.5pmol/L) with significantly elevated 1,25-dihydroxyvitamin D (1,25(OH)_2_D) of 335pmol/L (60-200pmol/L). Thyroid function tests, tumour markers, paraprotein screen, flow cytometry, ANCA antibodies and serum angiotensin converting enzyme were within normal range (Table 1). PTH-related-protein (PTHrP) was not available at our laboratory.

**Table 1:** Biochemistry results during initial admission

	Results	Reference range
Sodium	135mmol/L	135–145
Potassium	4.6mmol/L	3.2-5.0
Chloride	101mmol/L	95–110
Bicarbonate	27mmol/L	22–32
Urea	16.9mmol/L	3.0–7.0
Creatinine	286umol/L	45–90
Corrected calcium	3.52mmol/L	2.15–2.55
Phosphate	1.56mmol/L	0.75–1.50
Parathyroid hormone	1.2pmol/L	1.6–7.5
25-hydroxyvitamin D	101nmol/L	> 50
1,25-dihydroxyvitamin D	335pmol/L	60–200
Alkaline phosphatase	67 U/L	30–110
Angiotensin converting enzyme	64 U/L	20–70
Thyroid stimulating hormone	2.98 mIU/L	0.40-4.00
Total prostate specific antigen	0.75ug/L	< 4.50
ANCA – Myeloperoxidase Ab	< 0.2	< 1.0
ANCA – Proteinase 3 Ab	< 0.2	< 1.0

**Fig. 1:**
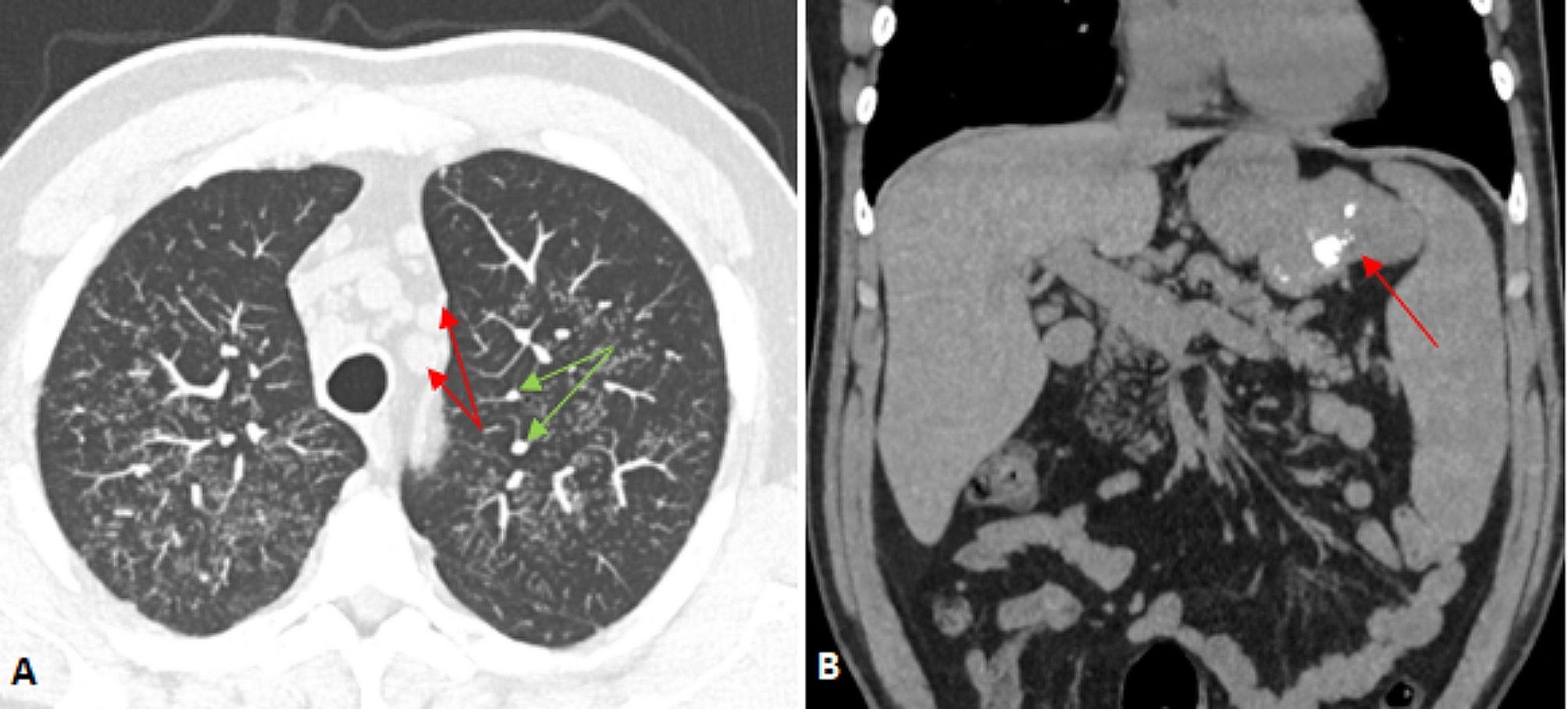
**(A)** Computed tomography chest demonstrating mediastinal lymphadenopathy (red arrows) and peri-lymphatic micronodules (green arrows) **(B)** Computed tomography abdomen demonstrating coalescent mass between stomach and spleen with calcification (red arrow)

Computed tomography (CT) of the chest, abdomen and pelvis demonstrated multiple lung micronodules, mediastinal lymphadenopathy and splenomegaly (18.3 cm) (Fig. 1A). However, an 11 × 6 cm coalescent mass with coarse calcification was also seen between the stomach and spleen (Fig. 1B). There were no osteolytic lesions seen.

**Fig. 2:**
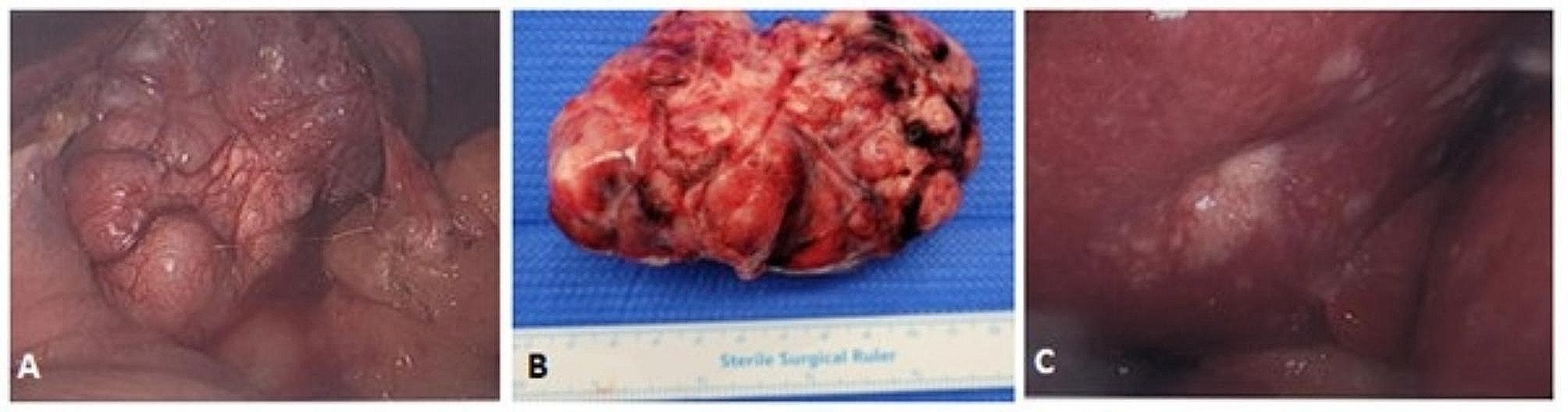
**(A)** Laparoscopic view of exophytic GIST arising from fundus of the stomach **(B)** Resected GIST **(C)** Nodular lesions on the liver

**Fig. 3:**
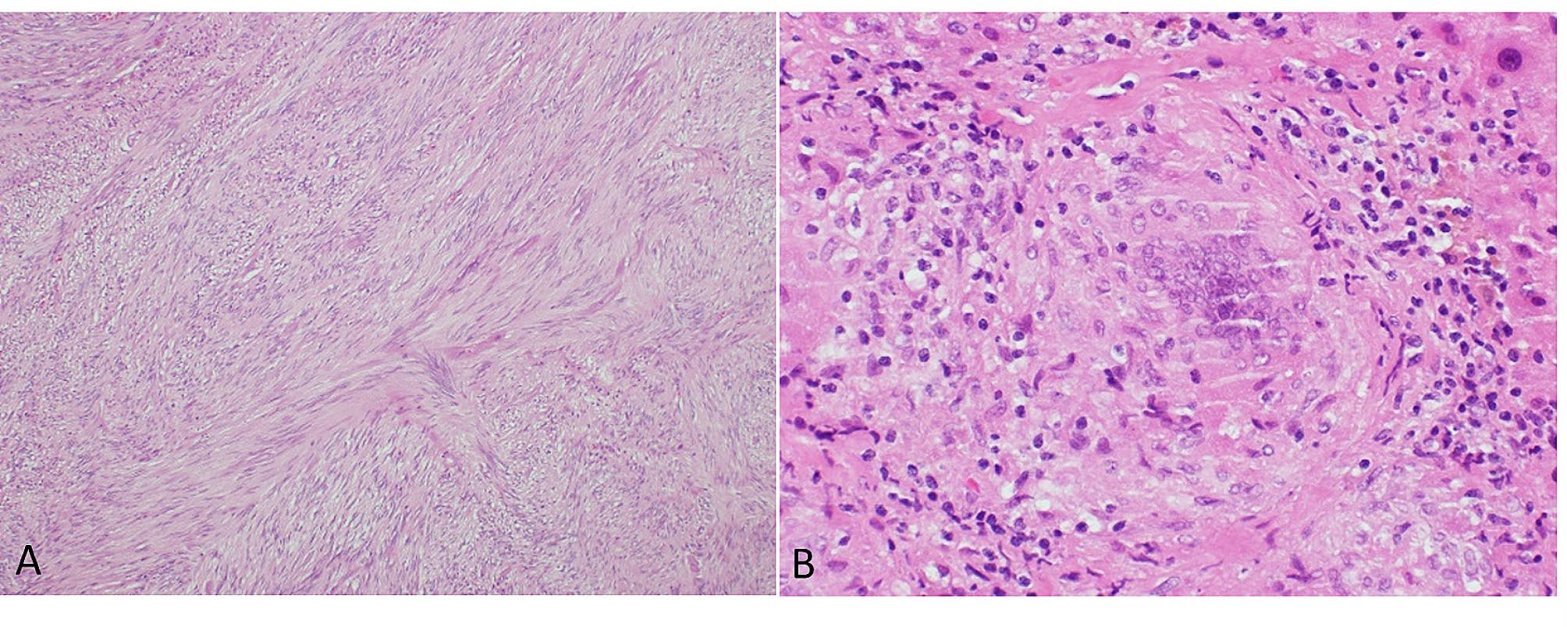
**(A)** Uniform spindle cell proliferation in fascicles and bundles, within the muscularis propria consistent with GIST **(B)** Non-caseating epithelioid granulomas centred upon portal tracts consistent with sarcoidosis

The patient proceeded to gastroscopy and laparoscopy, during which a 10 × 6 cm exophytic mass arising from the gastric fundus (Fig. 2A) was resected (Fig. 2B). Incidental nodular lesions of the liver were observed (Fig. 2C) and biopsied. Histopathology confirmed the exophytic mass to be a low-grade spindle-cell type gastrointestinal stromal tumour (GIST) (Fig. 3A) with positive staining for KIT proto-oncogene markers CD117 and DOG1, whilst liver biopsies showed non-necrotising epithelioid granulomas consistent with sarcoidosis (Fig. 3B). Mycobacteria were not seen on histopathology.

**Fig. 4:**
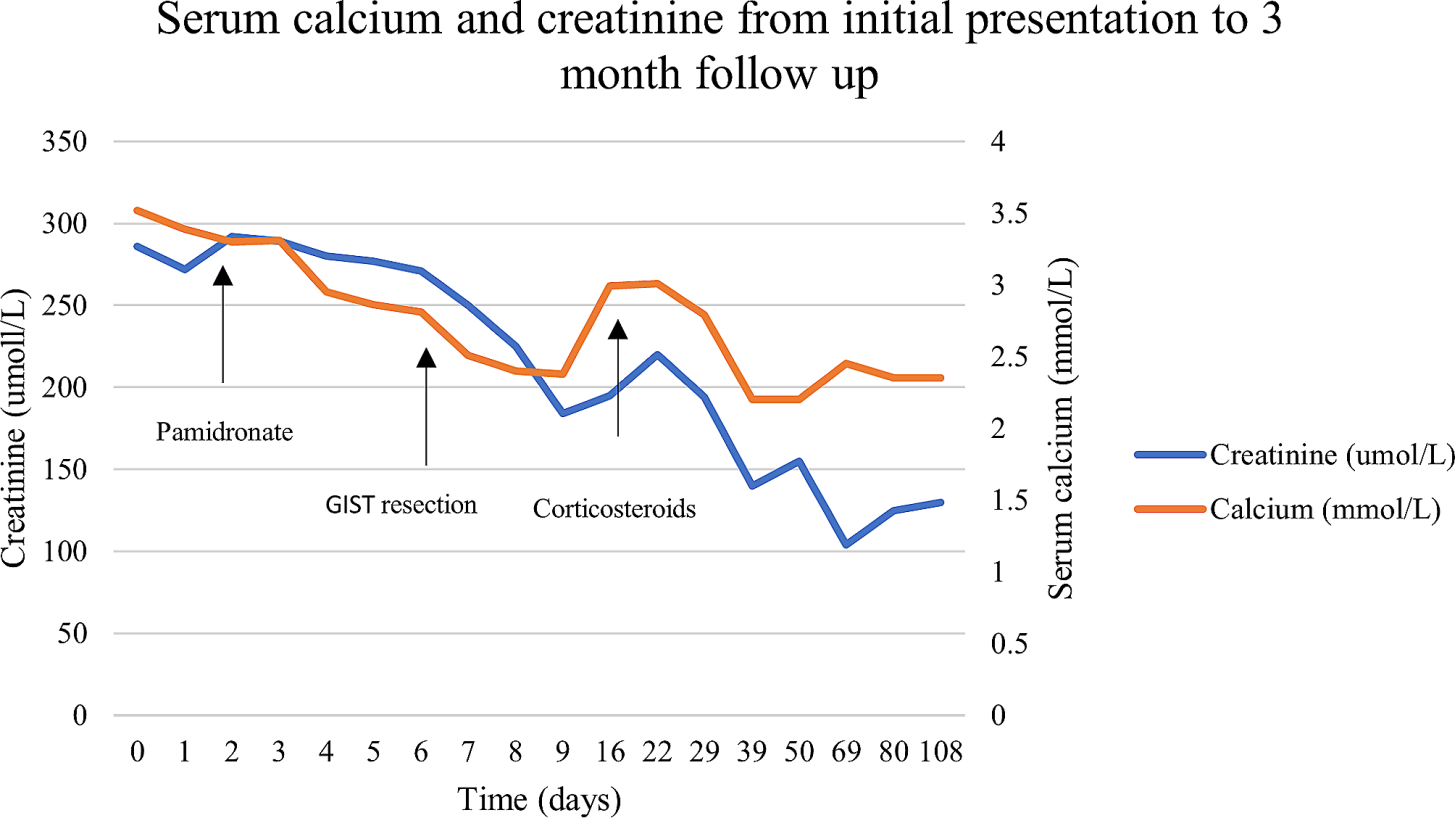
Serum calcium and creatinine from initial presentation to 3 month follow up

The patient was managed with intravenous fluids and pamidronate for hypercalcaemia. Following tumour resection, he was commenced on oral prednisone (1 mg/kg/day) for treatment of sarcoidosis with rapid resolution of his hypercalcaemia and acute kidney injury (Fig. 4). As the GIST showed low mitotic rate and surgical margins were clear, the risk of recurrence was deemed low (5–10%) and adjuvant therapy was not given. He was recommended for 6 to 12 monthly surveillance CT scans to monitor for recurrence.

At 3 months after initial diagnosis, the patient developed a painful, nodular rash over his right thigh. Interestingly, skin biopsy and tissue cultures revealed cutaneous infection with *Mycobacterium chelonae* without granulomatous response on histopathology. He was managed with clarithromycin and linezolid and his corticosteroid therapy was rapidly tapered by 4 months without recurrence of sarcoidosis at 9 months.

## Discussion

### GIST and hypercalcaemia

Malignancy-associated hypercalcaemia is mediated via three mechanisms: tumour secretion of PTHrP, metastatic disease to bone and over-production of 1,25(OH)_2_D [1]. Whilst PTHrP and bony metastases account for the majority of cases of malignancy-associated hypercalcaemia, in GISTs, the most common mechanism appears to be over-production of 1,25(OH)_2_D.

We found 11 case reports of GIST-associated hypercalcaemia in the literature with 6 cases documenting an elevation of 1,25(OH)_2_D [2–7]. High 1,25(OH)_2_D can arise from direct ectopic tumour secretion or from tumour production of 1-α hydroxylase, leading to the catalytic conversion of 25-hydroxyvitamin D (25(OH)D) to 1,25(OH)_2_D. The subsequent hyperabsorption of dietary calcium and increased renal tubular calcium reabsorption leads to hypercalcaemia.

Of the 11 case reports, elevated levels of PTHrP were observed in 2 cases [8, 9]. PTHrP functions similarly to PTH, increasing the synthesis of receptor activator of nuclear factor kappa (RANK) ligand and activating osteoclasts resulting in bone resorption and calcium release [1]. The true prevalence of PTHrP-mediated mechanisms for hypercalcaemia in GIST may be underreported owing to the variable availability of PTHrP testing at different sites, including ours. The remaining 3 case reports do not document a specific cause for hypercalcaemia [10–12].

GIST-associated metastatic bone disease occurs rarely and there are no case reports of this as the cause for hypercalcaemia [13]. Metastatic disease, however, is common; 9 out of 11 case reports we found reported metastatic disease at presentation, predominantly intraperitoneal, contrasting with the localized disease seen in our patient.

### GIST and sarcoidosis

Sarcoidosis is a multi-organ, granulomatous disease that commonly manifests as hypercalcemia and acute kidney injury, as in our case with presence of mediastinal lymphadenopathy and non-caseating granulomas on liver biopsy. It is a diagnosis of exclusion, and although interestingly this patient subsequently developed a mycobacterial infection whilst on high dose corticosteroids, mycobacterial, parasitic infection and malignancy were excluded initially by histopathology. Occupational exposures and drug-induced reactions were excluded on basis of history. Similar to malignancy-associated hypercalcaemia, mononuclear cells in granulomas express 1-α hydroxylase, catalysing the conversion of 25(OH)D into 1,25(OH)_2_D and leading to hypercalcaemia as previously described [14].

There is a clear association between sarcoidosis and malignancy; a meta-analysis by Bonifazi et al. of over 25,000 patients showed sarcoidosis to be associated with an increased risk of skin, haematopoietic, upper gastrointestinal, kidney, liver and colorectal cancers [15]. The pathophysiological link between the conditions, however, is less clear. Tumour-associated granulomas have been described in malignancies, whereby tumoral antigens derived from mutated peptides may induce immune activation and formation of granulomas through a T-cell medicated host response resulting in a sarcoid-like reaction [16]. Sarcoid-like reactions are histologically identical to that of sarcoidosis with non-caseating granulomas, and often occur in close proximity to the tumour or metastases [17]. In our case, although the liver biopsy showed non-caseating granulomas directly adjacent to GIST, the presence of concurrent mediastinal lymphadenopathy and hypercalcaemia which persisted after resection of the GIST, was consistent with systemic sarcoidosis.

The association between sarcoidosis and GISTs however, is less clear, with the current evidence limited to case series and case reports. Espejo et al., between 2007 and 2016, identified 8 patients with dual diagnoses of sarcoidosis and sarcomas in Florida (5 of the 8 cases being GISTs). The authors postulated that if the two conditions were independent, then these 8 cases represented a higher-than-expected rate of dual diagnoses, suggesting a non-random association [18]. Others have described individual case reports of dual diagnoses of GIST and sarcoidosis [19–21].

Our understanding of the potential pathophysiological relationship between the two conditions may also be hindered by the chronological variability in their detection. Of the cases we reviewed, sarcoidosis was detected *simultaneously with* GIST, *after* GIST and *before* GIST in approximately equal proportion (3 cases, 3 cases and 2 cases respectively) [18–21]. It is interesting to note that in Espejo et al.’s case series, in the cases where sarcoidosis was diagnosed *after* GIST, the sarcoidosis was diagnosed at the time of a GIST relapse [18]. Furthermore, owing to both conditions’ potential for insidious and often asymptomatic presentations, the time between the diagnoses of these conditions varied from 4 months up to 2.5 years, further clouding the ability to establish a clear chronological relationship.

### Clinical considerations

Despite unclear pathophysiological pathways, the growing number of case reports poses an interesting clinical question. When detected together, can definitive management of GIST assist in reducing the burden of corticosteroid therapy in the sarcoidosis? Although far from definitive, we found it notable that our patient’s corticosteroid therapy was rapidly truncated to 4 months due to the development of a mycobacterial infection and to date has not had a relapse of sarcoidosis.

We also wonder whether sarcoidosis could have concurrently been present in the previous case studies of GIST presenting with hypercalcaemia due to elevated 1,25(OH)_2_D levels. Without histopathology samples demonstrating granulomas, the biochemical features of sarcoidosis and non-sarcoidosis related hypercalcaemia in GIST may be difficult to distinguish and of note, at least 5 of the 6 studies with high 1,25(OH)_2_D levels also reported treatment of their patients with corticosteroids [2–4, 6, 7].

## Conclusion

Our case report contributes to the growing literature detailing dual diagnoses of GIST with sarcoidosis and is the first case report of GIST presenting with biopsy-proven sarcoidosis and hypercalcaemia. Further reporting is needed to establish a definitive connection between the two conditions and a common pathophysiologic pathway.

### Electronic supplementary material

Below is the link to the electronic supplementary material.

Supplementary Material 1

## Data Availability

All raw data generated during this study are included in the supplementary information file. The analysis of this data was used to construct a line graph which has been included in this article.

## Abbreviations

GIST: Gastrointestinal stromal tumour
PTH: Parathyroid hormone
PTHrP: Parathyroid hormone related protein
1,25(OH)2D: 1,25-dihydroxyvitamin D
25(OH)D: 25-hydroxyvitamin D
CT: Computed tomography
RANK: Receptor activator of nuclear factor kappa
